# Monoamine oxidase-dependent endoplasmic reticulum-mitochondria dysfunction and mast cell degranulation lead to adverse cardiac remodeling in diabetes

**DOI:** 10.1038/s41418-018-0071-1

**Published:** 2018-02-19

**Authors:** Soni Deshwal, Marleen Forkink, Chou-Hui Hu, Guido Buonincontri, Salvatore Antonucci, Moises Di Sante, Michael P Murphy, Nazareno Paolocci, Daria Mochly-Rosen, Thomas Krieg, Fabio Di Lisa, Nina Kaludercic

**Affiliations:** 1 Department of Biomedical Sciences, University of Padova, 35131 Padova, Italy; 2 Department of Medicine, University of Cambridge, Cambridge, CB2 0QQ UK; 3 Neuroscience Institute, National Research Council of Italy (CNR), 35131 Padova, Italy; 4 Mitochondrial Biology Unit, MRC, Cambridge, CB2 0XY UK; 5 Department of Medicine, Johns Hopkins Medical Institutions, Baltimore, MD 21205 USA; 6 Department of Experimental Medicine, University of Perugia, 06123 Perugia, Italy; 7 Department of Chemical and Systems Biology, School of Medicine, Stanford University, Stanford, CA 94305-5174 USA; 8 Present Address: Institute for Genetics, University of Cologne, Cologne, 50931 Germany

## Abstract

Monoamine oxidase (MAO) inhibitors ameliorate contractile function in diabetic animals, but the mechanisms remain unknown. Equally elusive is the interplay between the cardiomyocyte alterations induced by hyperglycemia and the accompanying inflammation. Here we show that exposure of primary cardiomyocytes to high glucose and pro-inflammatory stimuli leads to MAO-dependent increase in reactive oxygen species that causes permeability transition pore opening and mitochondrial dysfunction. These events occur upstream of endoplasmic reticulum (ER) stress and are abolished by the MAO inhibitor pargyline, highlighting the role of these flavoenzymes in the ER/mitochondria cross-talk. In vivo, streptozotocin administration to mice induced oxidative changes and ER stress in the heart, events that were abolished by pargyline. Moreover, MAO inhibition prevented both mast cell degranulation and altered collagen deposition, thereby normalizing diastolic function. Taken together, these results elucidate the mechanisms underlying MAO-induced damage in diabetic cardiomyopathy and provide novel evidence for the role of MAOs in inflammation and inter-organelle communication. MAO inhibitors may be considered as a therapeutic option for diabetic complications as well as for other disorders in which mast cell degranulation is a dominant phenomenon.

## Introduction

Cardiovascular complications account for the high morbidity and mortality in patients with type 1 and type 2 diabetes (T1D, T2D) [1]. Diabetic cardiomyopathy (DCM) is a distinct myocardial disease, characterized by structural changes in the heart and diastolic/systolic dysfunction. Several factors, including reactive oxygen species (ROS) formation and mitochondrial dysfunction, contribute to hyperglycemia-induced changes and to the etiology of DCM [2–7]. Indeed, respiratory chain-generated superoxide and p66^Shc^ lead to increased ROS levels characterizing mitochondria isolated from diabetic hearts or cardiomyocytes incubated with high glucose (HG) [8–12].

More recently, monoamine oxidases (MAOs) have been shown to play a major role in the oxidative stress and development of several cardiovascular pathologies, including DCM [13–16]. These flavoenzymes, localized at the outer mitochondrial membrane, exist as two isoenzymes (MAO-A and -B) and present unique features among ROS sources. The mechanism of catalysis has been elucidated and the crystal structure solved [17, 18]. During substrate degradation MAOs generate H_2_O_2_, aldehydes and ammonia [14], thus representing a source of ROS in the heart, particularly under stress conditions. In addition, MAOs utilize specific substrates important for cardiovascular pathophysiology, such as catecholamines and serotonin. Nevertheless, the mechanisms of MAO-induced cardiac damage are incompletely understood, especially in the context of DCM.

Endoplasmic reticulum (ER) stress is another key determinant in cardiac physiology and pathology [19, 20]. Impairment in ER homeostasis results in ER stress, accumulation of unfolded proteins and activation of complex signaling pathways collectively termed the unfolded protein response (UPR). If ER stress is severe or prolonged, the UPR may stimulate apoptosis, a significant feature of hypoxia, ischemia/reperfusion injury and DCM [19, 21]. Notably, ER and mitochondria are structurally and functionally connected [22–26]. Yet, whether mitochondrial ROS formation and dysfunction are upstream of ER stress or vice-versa is not known.

Inflammation exacerbates cellular responses to HG and, along with excessive ROS formation, is closely associated with tissue repair, scar formation, and fibrosis [27–29]. Cardiac resident mast cells hold secretory granules containing histamine, proteases and a variety of cytokines, growth factors, and other biologically active mediators capable of mediating tissue remodeling. Indeed, cardiac mast cell activation may contribute to fibrotic remodeling of the cardiac tissue [30, 31]. Although the pathologic importance of mast cells is becoming increasingly clear, triggers leading to their activation and degranulation have not been completely elucidated.

Despite the plethora of evidence that ROS formation, mitochondrial dysfunction and ER stress contribute to the development of DCM, the precise interplay between these events remains elusive. Here, we tested the hypothesis that MAO-induced ROS formation may represent the common denominator and investigated whether: (i) MAOs are involved in mitochondrial ROS formation and dysfunction triggered by hyperglycemia and inflammation; (ii) MAO-dependent mitochondrial derangements are upstream of ER stress occurring in the diabetic heart; (iii) MAO activity is related to cardiac fibrosis through mechanisms involving other cardiac cell types, such as mast cell degranulation.

## Results

### MAOs account for ROS formation in isolated cardiomyocytes exposed to HG and pro-inflammatory cytokine IL-1β

**Fig. 1:**
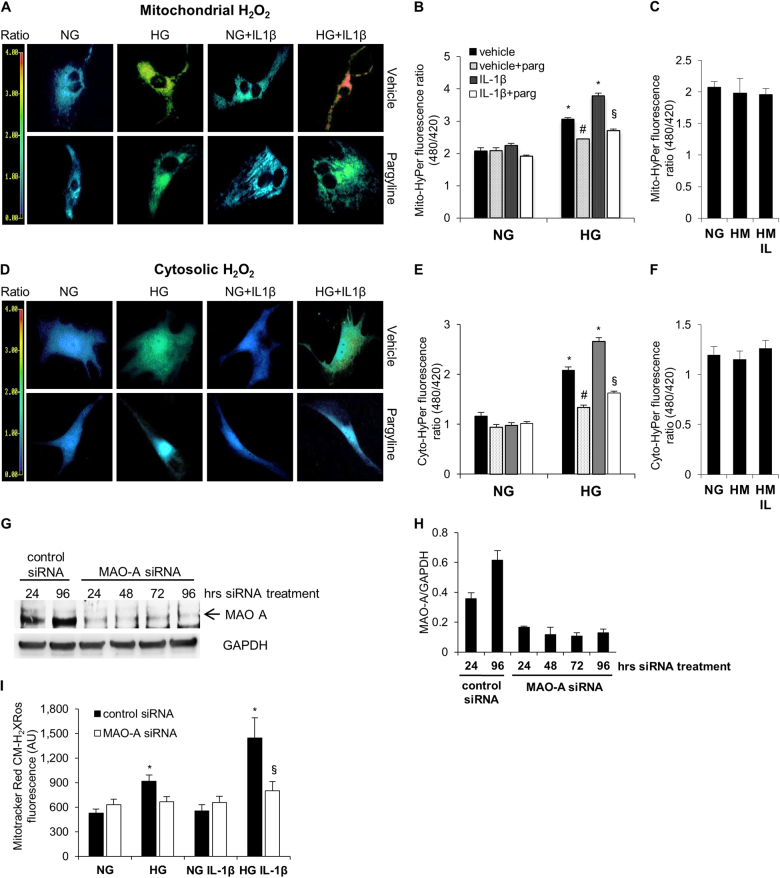
Effects of MAO inhibition on HG and pro-inflammatory cytokine IL-1β induced ROS formation in NRVMs. ROS formation was measured with genetically encoded H_2_O_2_ sensitive probe HyPer targeted either to mitochondria (**a**, **b**) or to the cytosol (**d**, **e**) upon NRVMs treatment with NG or HG in the absence or presence of IL-1β, and with or without pargyline. Effects of HM, as osmotic control, were assessed on mitochondrial (**c**) and cytosolic (**f**) H_2_O_2_ formation. MAO-A expression was assessed at different time-points following control or MAO-A siRNA treatment (**g**, **h**). ROS formation in control or MAO-A siRNA-treated cells was assessed upon treatment with NG or HG in the absence or presence of IL-1β (**i**). Approximately 100 cells were analyzed per condition in each experiment and all the experiments were performed at least three times. Data are expressed as mean ± SEM. Two-way ANOVA test followed by post hoc Tukey’s (**b** and **i**) or Dunn’s (**e**) multiple comparison test (*p < 0.05 vs NG vehicle, ^#^*p* < 0.05 vs HG vehicle and ^§^*p* < 0.05 vs HG-IL1β)

IL-1β, a pro-inflammatory cytokine and a regulator of the inflammatory response, is elevated in T1D and T2D [32]. Thus, to better mimic diabetic conditions in vitro, neonatal rat ventricular myocytes (NRVMs) were treated with HG alone or in combination with IL-1β. HG led to a significant increase in both mitochondrial (Fig. 1a, b) and cytosolic H_2_O_2_ formation after 48 h (Fig. 1d, e). This rise was not due to hyperosmolarity, since identical concentrations of mannitol did not show any changes in ROS levels (Fig. 1c, f, Supplementary Figures S1A and B). Moreover, IL-1β alone did not exert any effect on mitochondrial ROS formation at any of the time-points examined (Supplementary Figures 1C and D). Interestingly, combination of HG and IL-1β led to a further dramatic increase in H_2_O_2_ production when compared to HG or IL-1β treatment alone, indicating a synergistic effect of these stimuli in inducing ROS formation. Administration of pargyline, an inhibitor of both MAO isoforms, normalized ROS levels in these conditions (Fig. 1a–f), suggesting that HG and IL-1β induce ROS formation in a MAO-dependent manner. These results were confirmed using siRNA against MAO-A, the major MAO isoform expressed in the NRVMs (Supplementary Figures S2A and B). After 96 h of siRNA treatment, MAO-A protein expression was reduced by ~80% (Fig. 1g, h). HG and IL-1β were unable to induce an increase in ROS formation in these siRNA-treated cells, unequivocally demonstrating the contribution of MAO to this process (Fig. 1i).

**Fig. 2:**
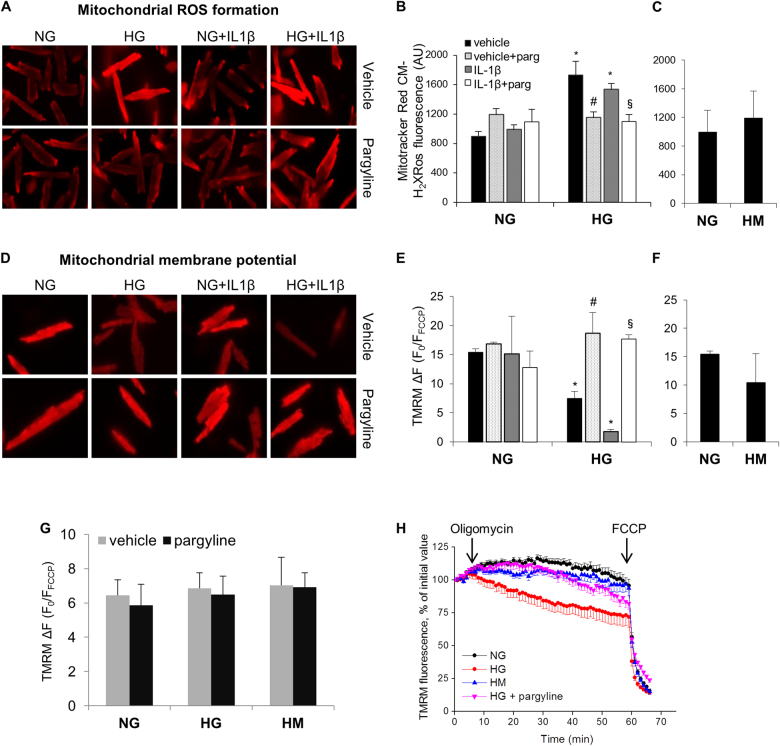
Effects of MAO inhibition on ROS formation and ΔΨ_m_ in cardiac myocytes exposed to HG and/or IL-1β. ROS formation was assessed with the fluorescent dye MTR in adult cardiomyocytes treated with NG or HG in the absence or presence of IL-1β, and with or without pargyline (**a**, **b**). Effects of the osmotic control HM on ROS formation (**c, f**). ΔΨ_m_ was measured in adult cardiomyocytes with the fluorescent dye TMRM in the same conditions as above (**d**–**f**). In neonatal cardiomyocytes, ΔΨ_m_ was measured following 48 h treatment with NG or HG in the absence or presence of IL-1β (**g**). Kinetic measurements were performed to follow TMRM fluorescence intensity following additions of oligomycin and FCCP, indicated by arrows (**h**). Approximately 100 cells were analyzed per condition in each experiment and all the experiments were performed three times. Data are expressed as mean ± SEM. Two-way ANOVA test followed by post hoc Tukey’s (**b**) or Dunn’s (**e**) multiple comparison test (*p < 0.05 vs NG vehicle, ^#^*p* < 0.05 vs HG and ^§^*p* < 0.05 vs HG + IL-1β vehicle)

To increase the translational value of the findings obtained in neonatal cells, we tested whether the same outcome holds true in the adult mouse cardiomyocytes. HG showed a more prominent effect in adult cardiomyocytes, leading to a ~2-fold increase in ROS generation after only 2 h of incubation (Fig. 2a, b). Again, this effect was not due to changes in osmotic pressure (Fig. 2c) or MAO protein abundance (Supplementary Figure S3). However, unlike NRVMs, no further increase in oxidative stress was evident after HG+IL-1β co-treatment. Pargyline treatment reduced ROS formation in both conditions, thus confirming that MAO plays a pivotal role in HG- and IL-1β-induced oxidative stress.

Since mitochondrial ROS have been described as inflammatory triggers [33], we investigated this link also in cardiomyocytes. Strong upregulation of NLRP3 and ASC upon lipopolysaccharide treatment confirmed that NRVMs are capable of activating NLRP3 inflammasome (Supplementary Figure S4A). Next, we assessed inflammasome activation in NRVMs treated with HG and/or IL-1β and observed an upregulation of NLRP3 only in cells treated either with IL-1β alone or with the combination of HG and IL-1β (Supplementary Figure 4B). This increase in NLRP3 expression was not affected in cells treated with pargyline, suggesting that MAO activity does not impact on inflammasome activation.

### MAO-dependent ROS generation impairs mitochondrial function in cardiomyocytes exposed to HG and IL-1β

Next, we investigated whether increased ROS emission due to HG and IL-1β, alone or in combination, is sufficient to alter mitochondrial function in cardiomyocytes. We found that mitochondrial membrane potential (ΔΨ_m_) was markedly reduced in adult cardiomyocytes already after 5 h of HG treatment (Fig. 2d–f). This effect was further exacerbated when HG was combined with IL-1β. Of note, there was no change in ΔΨ_m_ after 2 h of treatment with HG or HG+IL-1β (Supplementary Figure S5), suggesting that MAO-dependent ROS formation is upstream of mitochondrial (dys)function. Unlike adult cardiomyocytes, NRVMs exposed to HG did not show any changes in ΔΨ_m_ even after 48 h (Fig. 2g). These findings prompted us to examine whether HG induces latent mitochondrial dysfunction in NRVMs. It is well established that ATP synthase can mask the loss of ΔΨ_m_ by working in a reverse mode [34]. Thus, to assess whether ATP synthase activity was compensating for the dysfunctional respiratory chain in these cells, we monitored tetramethylrhodamine methyl ester (TMRM) fluorescence intensity in the presence of the ATP synthase inhibitor oligomycin. Normal glucose (NG) or high mannitol (HM) treated cells maintained ΔΨ_m_ for up to 1 h following oligomycin administration (Fig. 2h). On the other hand, TMRM fluorescence intensity started to drop immediately in HG-treated cells upon oligomycin addition. Hence, HG induces mitochondrial dysfunction in NRVMs, but in order to maintain ΔΨ_m_, these cells start to hydrolyze glycolytically synthesized ATP by reversing the activity of the ATP synthase. Pre-treatment with pargyline maintained ΔΨ_m_ in both neonatal and adult cardiomyocytes, indicating that MAO-generated ROS trigger mitochondrial dysfunction in cardiomyocytes treated with HG and IL-1β.

### MAO-generated ROS trigger mitochondrial depolarization through permeability transition pore opening

**Fig. 3:**
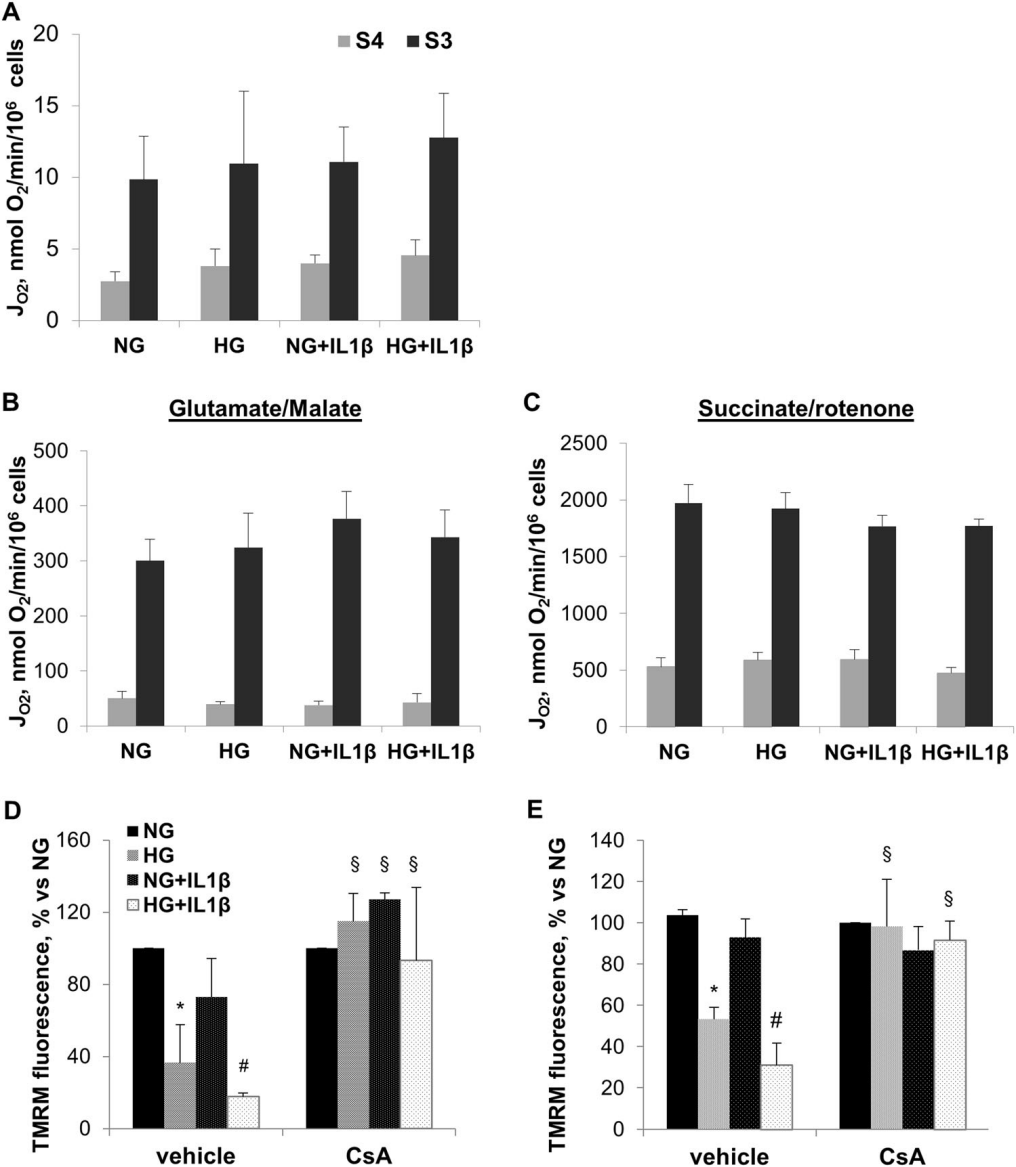
Mitochondrial respiration and PTP opening in cardiomyocytes exposed to HG and/or IL-1β. Oxygen consumption at baseline (S4) and following addition of ADP (S3) was measured in permeabilized neonatal (**a**, *n* = 3) or adult cardiomyocytes, using either glutamate/malate (**b**, *n* = 4) or succinate in the presence of rotenone (**c**, *n* = 3) as substrates. The effect of the PTP inhibitor CsA on ΔΨ_m_ was assessed in neonatal cardiomyocytes (**d**) upon 48 h treatment with NG or HG and IL-1β in presence of oligomycin. Adult cardiomyocytes (**e**) were treated with NG or HG and IL-1β for 5 h, in the presence and absence of CsA. For TMRM experiments, between 50 and 100 cells were analyzed per condition in each experiment and all the experiments were performed at least three times. Data are expressed as mean ± SEM. Two-way ANOVA test followed by post hoc Dunn’s multiple comparison test (*p < 0.005 vs NG, ^#^*p* < 0.05 vs NG + IL-1β, ^§^*p* < 0.005 vs respective group treated with vehicle)

To investigate the mechanism underlying mitochondrial dysfunction induced by MAO-generated ROS, we assessed the function of the respiratory chain by measuring oxygen consumption in permeabilized cells. Mitochondrial respiration was not impaired in any of the groups examined either in NRVMs (Fig. 3a) or in the adult cardiomyocytes (Fig. 3b, c), suggesting that the observed drop in ΔΨ_m_ was not due to alterations of the respiratory chain complexes. Considering that oxidative stress is a strong inducer of the permeability transition pore (PTP) opening [35], we next hypothesized that the loss of ΔΨ_m_ might have been caused by the opening of the PTP. Interestingly, mitochondrial depolarization following HG and/or IL-1β treatment was prevented when cells were co-treated with the PTP inhibitor cyclosporin A (CsA, Fig. 3d, e). These results suggest that MAO-dependent oxidative stress can directly target PTP to induce its opening thereby resulting in mitochondrial dysfunction.

### MAO inhibition prevents the activation of UPR in cardiomyocytes treated with HG and IL-1β

**Fig. 4:**
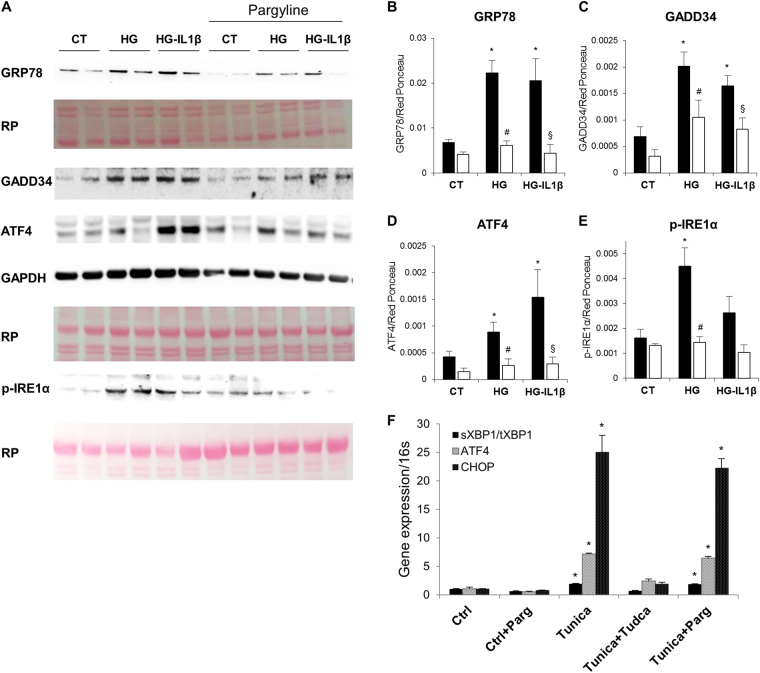
Effects of MAO inhibition on ER stress in adult cardiomyocytes exposed to HG and/or IL-1β. Representative western blots (**a**) and quantification of the protein expression of ER stress markers GRP78 (**b**), GADD34 (**c**), ATF4 (**d**), and phosphorylation levels of IRE1α (**e**) were assessed in adult cardiomyocytes treated with HG and IL-1β with (white) or without (black) pargyline for 48 h. Protein expression was normalized to total protein determined by Red Ponceau staining. Effects of the MAO inhibitor pargyline and the chemical chaperone TUDCA on tunicamycin-induced upregulation of ER stress markers (**f**, *n* = 3 per each group). All the experiments were performed at least three times. Data are expressed as mean ± SEM. Two-way ANOVA test followed by post hoc Tukey’s multiple comparison test (*p < 0.05 vs NG vehicle, ^#^*p* < 0.05 vs HG and ^§^*p* < 0.05 vs HG + IL-1β vehicle, ^†^*p* < 0.005 vs respective control, ^‡^*p* < 0.005 vs Tunica)

ER stress may be mediated by increased oxidative stress and vice-versa [22, 36, 37]. Here we sought to determine whether HG and IL-1β perturb ER function in a MAO-dependent manner. Expression levels of GRP78/BiP, an ER chaperone and a central regulator of UPR, were ~3 fold upregulated in the presence of HG and HG+IL1β (Fig. 4a, b). Furthermore, the expression of growth arrest- and DNA damage-inducible protein GADD34 (Fig. 4c) and transcription factor ATF4 (Fig. 4d), two proteins downstream of GRP78, was also significantly increased. Accordingly, phosphorylation levels of IRE1α, an ER transmembrane kinase, were higher as well, further confirming the activation of the UPR (Fig. 4e). Importantly, MAO inhibition prevented the UPR activation induced by HG and IL-1β in adult cardiomyocytes (Fig. 4a–e). Thus, the effects produced by MAO-dependent ROS formation go well beyond mitochondria, affecting also ER homeostasis to activate the UPR. Furthermore, excessive mitochondrial ROS formation and dysfunction are upstream of ER stress, at least under present experimental conditions. Next, we tested whether MAO takes part in the cell damage triggered by well-known ER stressors, such as tunicamycin and thapsigargin. The latter compounds led to severe ER stress that MAO inhibition was not able to abolish (Fig. 4f, Supplementary Figure S6A). Pargyline treatment partially reduced tunicamycin-induced ROS formation (Supplementary Figure S6B); however, it did not prevent cell death caused by either compound (Supplementary Figure S6C). These results show that MAO is central to ROS-induced ER stress/UPR activation. Accordingly, MAO inhibition effectively prevents ER stress when UPR is activated by oxidative stress, but not when ER stress is triggered by other redox-independent mechanisms, such as inhibition of N-linked glycosylation or depletion of ER Ca^2+^ stores.

### MAO inhibition prevents diastolic dysfunction in streptozotocin-treated mice

**Fig. 5:**
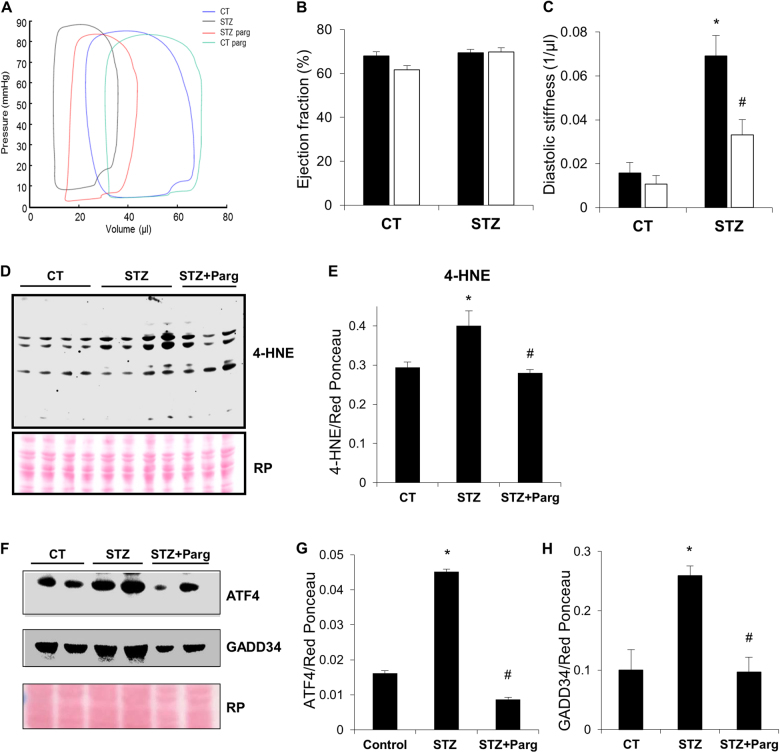
Characterization of LV function, oxidative and ER stress in vehicle- and pargyline-treated STZ-mice. Representative pressure-volume loops from control (blue, *n* = 6), STZ (black, *n* = 8), control + pargyline (green, *n* = 4) and STZ + pargyline (red, *n* = 10) treated mice (**a**). Ejection fraction (**b**) and diastolic stiffness (**c**) were determined in vehicle (black bars) or pargyline (white bars) treated control and STZ mice. Levels of 4-HNE (**d**, **e**) were assessed in heart tissue lysates from control, STZ and STZ+pargyline treated mice. Protein levels were normalized to total protein content determined by Red Ponceau staining (*n* = 5 per each group). Representative Western blot for ER stress markers performed in heart tissue lysates from control, STZ and STZ+pargyline treated mice (**f**). ATF4 (**g**) and GADD34 (**h**) band intensities were quantified and normalized to total protein content determined by Red Ponceau staining (*n* = 5 per each group). PV loop analysis was carried out by an observer blinded to the experimental conditions. Data are expressed as mean ± SEM. Two-way ANOVA test followed by post hoc Tukey’s multiple comparison test (*p < 0.05 vs CT vehicle, ^#^*p* < 0.05 vs STZ vehicle)

To investigate whether MAO contributes to the cardiac dysfunction associated with diabetes, MAO inhibitor pargyline was administered to streptozotocin (STZ) treated mice. Pressure-volume (PV) relationships revealed that STZ-mice have smaller hearts with a significant reduction in left ventricle (LV) chamber volumes (Fig. 5a and Table 1). STZ-mice treated with pargyline showed an improvement in cardiac morphology, although the differences were not statistically significant between the two groups. Heart rate and cardiac output were similar between groups (Table 1). We found that, although ejection fraction (an index of systolic function) was also unaffected in STZ-treated mice (Fig. 5b), dP/dt_max_ and dP/dt_min_ (load-dependent indices of contractility and relaxation) showed a trend to a decrease (Table 1). These data suggest that, whereas systolic function is unaffected after 12 weeks from diabetes induction, STZ mice can still develop overt systolic impairment with time. At the same time, there was an increase in diastolic stiffness, an index of diastolic dysfunction, which was 4.6-fold higher in the diabetic mice (Fig. 5c). Hence, our findings are congruent with several clinical reports showing that diastolic dysfunction is an early event in diabetic cardiomyopathy in which systolic impairment will eventually occur at later stages [7]. Importantly, pargyline administration prevented diastolic stiffening in STZ-mice, suggesting that MAO contributes to diastolic dysfunction in T1D.

**Table 1:** Glycemic and hemodynamic parameters measured in STZ-mice

	Control (*n* = 6)	Control + Parg (*n* = 4)	STZ (*n* = 8)	STZ + Parg (*n* = 10)
Glucose (mmol/l)	10.20 ± 1.40	8.60 ± 0.40	32.20 ± 30*	32.10 ± 3.10*
Body weight (g)	33.10 ± 2.70	32.40 ± 2.00	25.50 ± 4.30	24.20 ± 5.30
Heart rate (bpm)	380.56 ± 20.42	446.59 ± 21.90	350.57 ± 16.58	343.83 ± 1.94
EDV (µl)	65.66 ± 9.43	70.50 ± 2.60	47.62 ± 3.52*	50.70 ± 2.87*
ESV (µl)	21.50 ± 4.28	27.00 ± 1.41	14.87 ± 1.74	15.30 ± 1.49
SV (µl)	44.16 ± 5.31	43.50 ± 2.38	32.87 ± 2.12*	35.20 ± 2.11*
EF (%)	68.01 ± 2.06	61.75 ± 2.07	69.34 ± 1.76	69.82 ± 1.88
Diastolic stiffness (1/µl)	0.0158 ± 0.005	0.010 ± 0.004	0.069 ± 0.009*	0.0331 ± 0.007†
ESP (mm Hg)	82.66 ± 2.39	91.63 ± 5.72	67.47 ± 5.67	76.20 ± 1.94
EDP (mm Hg)	8.58 ± 1.53	5.98 ± 1.79	9.21 ± 2.20	10.32 ± 1.75
dP/dt_max_ (mm Hg/s)	5487.40 ± 430.94	7120.29 ± 522.16	4101.85 ± 645.78	4713.28 ± 316.7
dP/dt_min_ (mm Hg/s)	−5930.80 ± 389.2	−7161.70 ± 804.36	−4001.3 ± 886.9	−4500.29 ± 885.7
Efficiency (%)	73.35 ± 4.15	70.37 ± 17.02	76.22 ± 6.62	74.06 ± 5.36

*EDV* end-diastolic volume, *ESV* end-systolic volume, *SV* stroke volume, *EF* ejection fraction, *CO* cardiac output, *ESP* end-systolic pressure, *EDP* end-diastolic pressure. Data are expressed as mean ± SEM. Two-way ANOVA test followed by post hoc Tukey’s test (*p < 0.005 vs Control, ^†^*p* < 0.005 vs STZ).

### Oxidative stress and ER stress are reduced in STZ hearts upon pargyline treatment

We next examined whether MAO contributes to oxidative and ER stress in T1D in vivo. Formation of 4-hydroxynonenal (4-HNE), an aldehyde product of lipid peroxidation, was measured as an index of oxidative stress. In line with our in vitro data, we found increased levels of 4-HNE in STZ hearts (Fig. 5d, e). This was accompanied by impaired ER homeostasis as demonstrated by the induction of UPR associated proteins ATF4 (Fig. 5f, g) and GADD34 (Fig. 5f, h). Pargyline administration to diabetic mice completely prevented these alterations, suggesting that hyperglycemia-induced changes in diabetic hearts lead to enhanced MAO-mediated ROS generation, eventually prompting cardiac redox imbalance and activation of the UPR.

### MAO activity triggers cardiac fibrosis and mast cell degranulation in STZ hearts

**Fig. 6:**
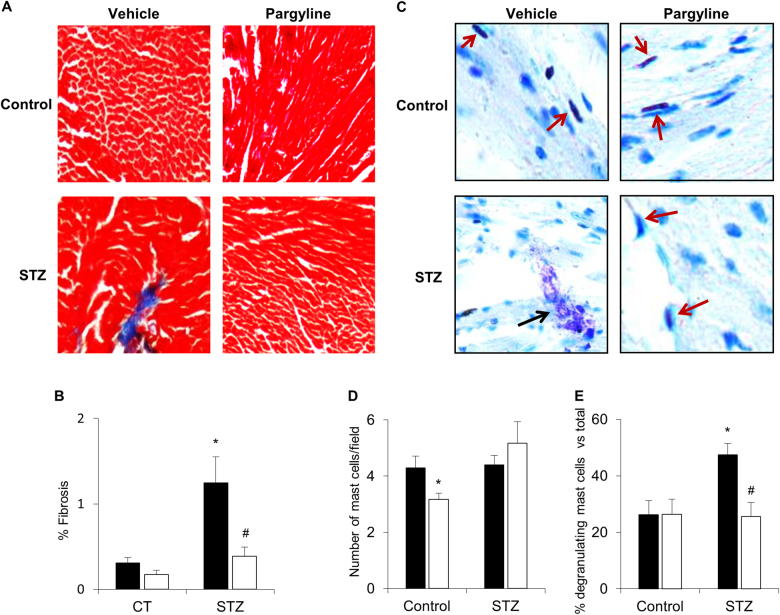
Effects of MAO inhibition on cardiac fibrosis and mast cell degranulation in STZ-mice. Representative images of Masson’s trichrome staining from control and STZ mice, showing collagen deposition in blue (**a**). The quantification data are expressed as percentage of fibrotic vs total cardiac tissue (**b**). Black and white bars represent vehicle and pargyline treated mice, respectively (Control *n* = 10, control+pargyline *n* = 5, STZ *n* = 12, STZ+pargyline *n* = 15). Representative images of toluidine blue staining of cardiac tissue from indicated experimental groups (**c**). Red arrows indicate intact mast cells, and black arrows indicate actively degranulating mast cells. Quantification of mast cell density (**d**) and mast cell degranulation (**e**). Results are expressed as number of mast cells per field and percentage of degranulating mast cells vs total number of mast cells, respectively. (Control *n* = 10, control+pargyline *n* = 5, STZ *n* = 12, STZ+pargyline *n* = 15). Analysis was carried out by an observer blinded to the experimental conditions. Data are expressed as mean ± SEM. Two-way ANOVA test followed by post hoc Tukey’s (**d** and **e**) or Dunn’s (**b**) multiple comparison test (*p < 0.05 vs control vehicle, ^#^*p* < 0.05 vs STZ vehicle)

Cardiac fibrosis is one of the underlying causes of diastolic dysfunction and a major feature of DCM [2, 38]. We observed that STZ-treated hearts displayed 4-fold increase in collagen deposition as compared to normal hearts (Fig. 6a, b). Interestingly, pargyline treatment abrogated this change, demonstrating MAO inhibitors’ ability to prevent fibrosis progression in T1D animals. Since mast cell degranulation is involved in fibrosis development and known to play a key role in the inflammatory process by releasing a number of pro-inflammatory and pro-fibrotic factors [31, 39], we next assessed cardiac mast cell density and level of degranulation in STZ vs control mice (Fig. 6c). Although mast cell density was similar between the groups (Fig. 6d), the extent of degranulation was markedly higher in STZ mice (Fig. 6e). Pargyline treatment prevented this event in T1D mice. Thus, MAO-induced ROS production can trigger cardiac mast cell activation and degranulation. This phenomenon, in turn, contributes to the remodeling of the extracellular matrix, ultimately resulting in LV fibrosis and dysfunction.

## Discussion

**Fig. 7:**
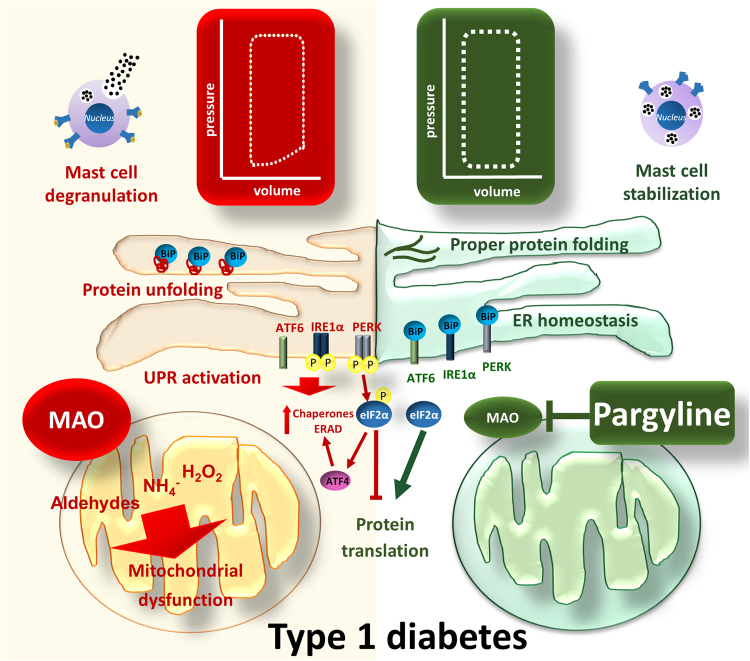
Schematic representation of the effects elicited by MAO-dependent oxidative stress in diabetic cardiomyopathy. Hyperglycemia and pro-inflammatory stimuli result in enhanced mitochondrial MAO-dependent H_2_O_2_ formation that, in turn, targets mitochondria to induce mitochondrial dysfunction. These events occur upstream of the impairment in ER homeostasis and accumulation of unfolded proteins in the ER lumen. The consequent activation of the UPR through three ER stress branches, namely IRE1α, PERK and ATF6, leads to protein translation attenuation, upregulation of chaperones and activation of ERAD machinery. Moreover, MAO-dependent oxidative stress in vivo contributes to mast cell degranulation and cardiac fibrosis, ultimately resulting in diastolic dysfunction in type 1 diabetes. Administration of the MAO inhibitor pargyline prevents exacerbated ROS formation and restores mitochondrial and ER homeostasis. In addition, MAO inhibition abolishes mast cell degranulation and fibrosis, thus improving LV diastolic function. *ATF4* activation transcription factor 4, *ATF6* activation transcription factor 6, *BiP* binding immunoglobulin protein, *ER* endoplasmic reticulum, *ERAD* ER associated degradation, *eIF2α* eukaryotic initiation factor 2α, *IRE1α* inositol-requiring enzyme 1α, *IL-1β* interleukin 1β, *MAO* monoamine oxidase, *PERK* protein kinase R-like endoplasmic reticulum kinase, *ROS* reactive oxygen species, *UPR* unfolded protein response

The present results demonstrate that MAO-induced ROS formation causes mitochondrial dysfunction and ER stress, factors that ultimately promote DCM development (Fig. 7). The improvement in diastolic stiffness elicited by MAO inhibition in vivo is accompanied by a reduction in interstitial fibrosis, establishing a previously unappreciated mechanistic link between cardiac fibrosis and mast cell degranulation that is MAO-dependent.

Many alterations in cellular and mitochondrial metabolism observed during the development of DCM are associated with increased ROS levels. In this setting, cardiac impairment is not caused by hyperglycemia per se; rather, oxidative stress and inflammation lead to cardiovascular complications. Mitochondrial ROS formation and dysfunction lead to the production of IL-1β via the inflammasome pathway [33]. In our setting, NLRP3 inflammasome was hardly activated and occurred independently of MAO-induced mitochondrial ROS formation. Indeed, HG does not lead to inflammasome activation in NRVMs [40], suggesting that cardiomyocytes are a target rather than a source of IL-1β and other inflammatory stimuli produced by other cells, such as macrophages. Yet, whether and how IL-1β can induce mitochondrial ROS formation has not been reported. Other studies have shown that diabetes-induced mitochondrial dysfunction and ROS formation occur through calpain [41] and O-linked β-N-acetylglucosamine glycosylation [42]. Here we show that IL-1β, in combination with HG, induces mitochondrial ROS formation and dysfunction in a MAO-dependent manner. We further demonstrate that the opening of the PTP underlies MAO-induced mitochondrial depolarization. This finding supports the tight link between oxidative stress and PTP opening and indicates MAO activity as an upstream event or part of an amplification pathway exacerbating mitochondrial dysfunction. Future investigations will address whether and how MAO activity impacts on F_O_F_1_ ATPase that has been recently described as the molecular identity of the PTP [43, 44].

Hyperglycemia and inflammation induce ER stress and consequently trigger the UPR [40, 45]. Severe and/or prolonged ER stress leads to mitochondrial dysfunction, impaired Ca^2+^ and redox homeostasis, thus playing a predominant role in the pathogenesis of multiple cardiovascular diseases [19, 22, 37]. However, the opposite also holds true. In fact, aberrant ROS formation and alterations in mitochondrial structure and function may affect ER homeostasis [36]. Here we show that MAO activity primarily accounts for impaired ER homeostasis and activation of the UPR. Moreover, HG- and inflammation-induced ROS formation and mitochondrial dysfunction in cardiomyocytes occur upstream of ER stress, given that abrogation of MAO-dependent ROS formation was sufficient to abolish the UPR in those conditions. These findings highlight a novel role for these flavoenzymes as a key linchpin between excessive ROS formation and ER stress. In addition, our present data show that MAO inhibition was not sufficient to protect cells from damage when ER stress was induced directly and occurred upstream of mitochondrial ROS formation. This outcome is likely due to the fact that both tunicamycin and thapsigargin lead to a more severe ER stress in comparison to our in vitro diabetic model. On the other hand, it is plausible that tunicamycin- and thapsigargin-induced ER stress causes mitochondrial dysfunction, oxidative stress and cell death in a MAO-independent manner. Indeed, ER stress can induce cell death as a consequence of SR Ca^2+^ release and PTP opening [46].

Another study identified MAO-A as an important source of ROS in STZ-treated rats, suggesting that its inhibition may improve cardiac contractility [16]. These Authors focused on the later stages of DCM, characterized by reduced heart rate and contractility, and likely associated with reduced ejection fraction and dilation. However, most recent clinical reports on DCM describe a phenotype that differs from dilated cardiomyopathy [47]. The typical diabetic patient suffering from DCM falls into the category of heart failure with preserved ejection fraction (HFpEF), presenting a small LV cavity, normal systolic LV ejection fraction, thick LV walls, elevated LV filling pressures and diastolic dysfunction [47]. Therefore, it is important to assess whether MAOs contribute to the early impairment in diabetic hearts, represented by diastolic dysfunction. Here we provide unprecedented evidence that diastolic stiffness observed in STZ mice is prevented by MAO inhibition. These findings are of major clinical relevance because several heart failure drugs showed positive outcomes in heart failure patients with reduced ejection fraction, but the outcome was neutral in HFpEF [47]. Currently, treatment of DCM with HFpEF phenotype is limited to diuretics and lifestyle modification. Therefore, our results highlight the therapeutic potential of MAO inhibitors in the latter cohort of diabetic/HFpEF patients, while providing key mechanistic advancement concerning the mechanisms underlying MAO-induced changes in the diabetic heart. Future studies must be designed ad hoc to test whether MAO inhibition affords benefits even when instituted after the onset of diastolic dysfunction, i.e., at later stages of DCM.

Collagen deposition and extracellular matrix remodeling result in the stiffening of the cardiac tissue and diastolic dysfunction. Mast cell degranulation is a key-contributing factor in heart failure pathogenesis, irrespective of the etiology [31]. However, the exact mechanisms leading to mast cell degranulation are unclear, partly owing to the multiplicity of environmental stressors they are sensitive to. Oxidative stress is a trigger for mast cell activation [48], but until now no study has identified specific ROS source liable for mast cell degranulation in DCM. Here we report that MAO activity triggers mast cell degranulation in STZ-mice, thus providing a mechanistic link between these mitochondrial flavoenzymes, inflammation and fibrosis in DCM. Interestingly, mast cell stabilizer nedocromil has been shown to only partially prevent structural and functional changes in the diabetic heart [39]. It is therefore tempting to hypothesize that the stronger protection observed in our study is due to the synergistic effects of MAO inhibition on cardiomyocytes and mast cells. Further studies are necessary to elucidate the exact mechanisms linking MAO activation and mast cell degranulation.

Besides H_2_O_2_ formation, MAOs are also a source of reactive aldehydes and ammonia [14]. Stimulation of mitochondrial ALDH2 activity, the enzyme responsible for aldehyde conversion into the corresponding carboxylic acids, improves mitochondrial function and reduces cardiac damage in several models of cardiac injury, including T1D [49]. It is plausible that MAO inhibition in diabetes abolishes the detrimental effects of these reactive aldehydes as well. Another aspect of our study that warrants further, in-depth investigation is the exact mechanism leading to the upregulation of MAO activity in diabetes. Although we did not observe changes in MAO protein expression (Supplementary Figures S3 and S7), increased substrate availability, post-translational modifications or interactions with other cellular pathways might account for its increased activity. In that regard, we have excluded that oxidation may cause changes in MAO activity (Supplementary Figure S8). Our data suggest that MAO activity is either absent or negligible at baseline and becomes relevant only upon pathological stimuli, most likely when substrate availability is increased. This is in line with our previous observations showing that, despite protein levels remained unchanged, augmented availability and degradation of MAO substrates norepinephrine and dopamine triggered maladaptive signaling pathways leading to hypertrophy and heart failure in pressure-overloaded mice [13, 15]. In addition, histamine has been shown to trigger MAO-dependent impairment in vasorelaxation [50], although the signaling pathway linking histamine receptors and/or metabolism with MAO activity was not elucidated. Considering that histamine, dopamine and serotonin are all contained in mast cell granules, it is possible that these substrates are responsible for MAO activation, at least in this cell type. However, MAOs degrade many other biogenic amines as well; thus, identification of substrates responsible for increased MAO activity in diabetic conditions warrants further investigation involving a metabolomics approach.

In conclusion, the present findings identify MAOs as major vehicles of damage during the hyperglycemic and pro-inflammatory states characterizing DCM. By extension, MAO inhibitors that are currently used in the clinic for treating depression or Parkinson’s disease should be considered as a therapeutic option for diabetes and its complications as well as for other disorders in which mast cell degranulation is a dominant phenomenon.

## Materials and methods

### Animals

All animal studies were performed using male C57BL6/J mice (6–7 weeks of age; Charles River Laboratories, UK). T1D was induced with STZ intraperitoneally for five consecutive days (50 mg/kg/day in citrate buffer pH 4.5). Animals were then randomized and treated either with vehicle or MAO inhibitor pargyline (50 mg/kg/day). Blood glucose levels were measured twice a month using glucose meter (OneTouch Ultra 2) and mice with blood glucose levels ≥17 mM were considered diabetic. Animal studies were performed in accordance with relevant guidelines and regulations and were approved by the Italian Ministry of Health and the University of Cambridge.

### In vivo cardiac function

In vivo cardiac function was assessed using MRI-calibrated PV loops. First, a MRI scan was performed as previously described to assess systolic function [51, 52]. Mice were quickly anaesthetized with isoflurane followed by a 70 mg/kg intraperitoneal injection of pentobarbital. After endotracheal intubation for mechanical ventilation (Harvard apparatus, Holliston, MA), mice underwent closed-chest catheterization of the left ventricle via the carotid artery. PV data was acquired using a Transonic ADV 500 system (iWorx 125 Systems Inc., NH). Before PV loop analysis, volumes were smoothed (smoothing kernel of 4 ms), and calibrated using the MRI data. The maximum and minimum volumes obtained with the catheter were matched to the MRI-derived LV volumes. Summary pressure and diastolic stiffness values were derived using Labscribe 2 (iWorx 125 Systems Inc., NH).

### Primary myocytes studies

NRVMs were isolated from 1 to 3 days old rats, as previously described [15] and plated at the density of 100,000 cells/ml in minimum essential media (MEM) supplemented with FBS 10%, 0.1 mM BrdU, antibiotics, and non-essential amino acids. Cells were maintained at 37°C, 5% CO_2_. The medium was changed to MEM supplemented with 1% FBS after 24 h. Adult mouse ventricular myocytes were isolated from the hearts of 12 weeks old C57Bl6/J mice, as previously described [53]. Collagenase-digested isolated myocytes were incubated in buffer with increasing concentrations of Ca^2+^, achieving a final concentration of 1.2 mM Ca^2+^ as in MEM culture media (Life Technologies). Cells were seeded at 25,000 rod-shaped myocytes/ml on coverslips coated with laminin. After 1 h incubation at 37°C, 2% CO_2_, culture media was replaced to remove unattached cells.

ROS formation in NRVMs was measured using the genetically encoded H_2_O_2_ sensitive mito-HyPer or cyto-HyPer plasmids (Evrogen) or 25 nM Mitotracker Red (MTR, CM-H_2_XRos, Life Technologies). Cells were transfected with the calcium phosphate method and treated for 48 h in following conditions: NG 5 mM, HG 25 mM or HM 25 mM in the presence or absence of 25 ng/ml rat IL-1β (Sigma). To inhibit MAO activity cells were either pre-treated with 100 µM pargyline (Sigma) or transfected with scramble or MAO-A siRNA (Ambion). Adult cardiomyocytes were loaded with 250 nM MTR, washed and treated as above for 2 h. Images were captured using fluorescence microscope (Leica). Analysis and fluorescence intensities were determined using computer-assisted image analysis systems (ImageJ, NIH).

To measure ΔΨ_m_, cardiomyocytes were incubated with NG, HG and IL-1β as above in the absence or presence of pargyline or CsA (1 µg/ml) for 5 h and then loaded with 25 nM TMRM, a cell-permeant dye sequestered by active mitochondria, in the presence of 1.6 µM cyclosporin H. Images were collected using a fluorescence microscope (Leica) before and after the addition of 6 µM oligomycin (to NRVMs only) and 2.5 µM FCCP. Images were analyzed and fluorescence intensities determined using computer-assisted image analysis systems (ImageJ, NIH). Results are expressed as ΔF (F_0_/F_FCCP_).

To measure oxygen consumption, cells were detached and permeabilized with 10 µM saponin in the respiration medium (125 mM sucrose, 65 mM KCl, 20 mM Hepes, 1 mM MgCl_2_, 200 µM EGTA, 2.5 mM KH_2_PO_4_). Next, cells were added to the respiration chamber and basal (state 4) respiration was monitored following addition of respiration substrates, 5/2.5 mM glutamate/malate (complex I) or 5 mM succinate in the presence of 2 µM rotenone (complex II). After 3 min, 1 mM ADP was added to induce state 3 respiration. Data are expressed as the rate of oxygen consumption normalized to the number of cells used for the assay.

### MAO activity

MAO activity was measured fluorometrically in permeabilized cells in an assay using 10-acetyl-3,7-dihyrdoxyphenoxazine (Amplex Red reagent, Life Technologies), in combination with horseradish peroxidase (HRP), to detect H_2_O_2_ generated during substrate catabolism. Following exposure to H_2_O_2_, recombinant MAO-A and –B (Sigma) were spun down and resuspended in PBS. MAO protein (3.5 µg/reaction) was diluted in PBS containing 10 µM Amplex Red and 7.5 µg/ml HRP and added into black opaque 96-well microplates. Amplex Red fluorescence was measured kinetically with a fluorescence microplate reader (Fluoroskan Ascent Microplate fluorometer, Thermo Fisher Scientific, excitation/emission wavelengths 560/590 nm), at baseline and following addition of substrates. MAO-A and -B activities were assayed using tyramine and phenylethylamine as substrates, respectively. The velocity of H_2_O_2_ production was calculated from a calibration curve obtained adding known amounts of H_2_O_2_ and results were expressed as rate of H_2_O_2_ formation per µg protein.

### Histology

Hearts were fixed in 10% formalin overnight, embedded in paraffin and sectioned at 5 µm thickness. To detect collagen deposition, sections were stained with Masson’s Trichrome (Bio-Optica, Milan, Italy). Photomicrographs of the sections were evaluated for interstitial collagen fractions using computer-assisted image analysis systems (Adobe Photoshop).

Metachromatic staining with toluidine blue (0.1%, pH 2.3, for 3 min) was employed to detect mast cell density and degranulation [31]. Mast cell density was determined counting the total number of mast cells per field. Mast cell degranulation was expressed as the number of degranulating mast cells normalized to total number of mast cells.

### Real time-PCR

Total RNA was extracted from NRVMs using Trizol reagent (Invitrogen). Isolated RNA was reverse transcribed to cDNA using random hexamers, dNTPs and SuperScript III reverse transcriptase (Invitrogen), following manufacturer’s protocol and amplified by PCR.

### Western blot

Cardiomyocytes or heart tissue were homogenized in lysis buffer containing protease and phosphatase inhibitors. Protein concentration was determined using BCA protein assay (Pierce). Proteins were separated using SDS–PAGE (Invitrogen) and transferred to nitrocellulose membrane (Bio-Rad). Following incubation with primary and secondary HRP-conjugated antibody (Bio-Rad), bands were detected using KODAK Image station 4000 MM PRO and analyzed using Gel-Pro software.

### Statistical analysis

All values are expressed as mean±SEM. Comparison between groups was performed by one-way or two-way ANOVA, followed by Tukey’s post hoc multiple comparison for normally distributed data and non-parametric Dunn’s test for not normally distributed data. Comparisons between two groups were performed using non-paired two-tailed Student’s *t*-test. A value of *p* < 0.05 was considered significant.

## Electronic supplementary material

Supplementary Figures

## References

[1] Grundy SM, Benjamin IJ, Burke GL, Chait A, Eckel RH, Howard BV (1999). Diabetes and cardiovascular disease: a statement for healthcare professionals from the American Heart Association. Circulation..

[2] Bugger H, Abel ED (2014). Molecular mechanisms of diabetic cardiomyopathy. Diabetologia..

[3] Baseler WA, Dabkowski ER, Jagannathan R, Thapa D, Nichols CE, Shepherd DL (2013). Reversal of mitochondrial proteomic loss in Type 1 diabetic heart with overexpression of phospholipid hydroperoxide glutathione peroxidase. Am J Physiol Regul Integr Comp Physiol..

[4] Matsushima S, Kinugawa S, Ide T, Matsusaka H, Inoue N, Ohta Y (2006). Overexpression of glutathione peroxidase attenuates myocardial remodeling and preserves diastolic function in diabetic heart. Am J Physiol Heart Circ Physiol..

[5] Ye G, Metreveli NS, Donthi RV, Xia S, Xu M, Carlson EC (2004). Catalase protects cardiomyocyte function in models of type 1 and type 2 diabetes. Diabetes..

[6] Shen X, Zheng S, Metreveli NS, Epstein PN (2006). Protection of cardiac mitochondria by overexpression of MnSOD reduces diabetic cardiomyopathy. Diabetes..

[7] Boudina S, Abel ED (2010). Diabetic cardiomyopathy, causes and effects. Rev Endocr Metab Disord..

[8] Nishikawa T, Edelstein D, Du XL, Yamagishi S, Matsumura T, Kaneda Y (2000). Normalizing mitochondrial superoxide production blocks three pathways of hyperglycaemic damage. Nature..

[9] Di Lisa F, Giorgio M, Ferdinandy P, Schulz R (2017). New aspects of p66Shc in ischaemia reperfusion injury and other cardiovascular diseases. Br J Pharmacol..

[10] Shen E, Li Y, Li Y, Shan L, Zhu H, Feng Q (2009). Rac1 is required for cardiomyocyte apoptosis during hyperglycemia. Diabetes..

[11] Dabkowski ER, Williamson CL, Bukowski VC, Chapman RS, Leonard SS, Peer CJ (2009). Diabetic cardiomyopathy-associated dysfunction in spatially distinct mitochondrial subpopulations. Am J Physiol Heart Circ Physiol..

[12] Boudina S, Sena S, Theobald H, Sheng X, Wright JJ, Hu XX (2007). Mitochondrial energetics in the heart in obesity-related diabetes: direct evidence for increased uncoupled respiration and activation of uncoupling proteins. Diabetes..

[13] Kaludercic N, Carpi A, Nagayama T, Sivakumaran V, Zhu G, Lai EW (2014). Monoamine oxidase B prompts mitochondrial and cardiac dysfunction in pressure overloaded hearts. Antioxid Redox Signal..

[14] Kaludercic N, Mialet-Perez J, Paolocci N, Parini A, Di Lisa F (2014). Monoamine oxidases as sources of oxidants in the heart. J Mol Cell Cardiol..

[15] Kaludercic N, Takimoto E, Nagayama T, Feng N, Lai EW, Bedja D (2010). Monoamine oxidase A-mediated enhanced catabolism of norepinephrine contributes to adverse remodeling and pump failure in hearts with pressure overload. Circ Res..

[16] Umbarkar P, Singh S, Arkat S, Bodhankar SL, Lohidasan S, Sitasawad SL (2015). Monoamine oxidase-A is an important source of oxidative stress and promotes cardiac dysfunction, apoptosis, and fibrosis in diabetic cardiomyopathy. Free Radic Biol Med..

[17] Binda C, Newton-Vinson P, Hubalek F, Edmondson DE, Mattevi A (2002). Structure of human monoamine oxidase B, a drug target for the treatment of neurological disorders. Nat Struct Biol..

[18] Son SY, Ma J, Kondou Y, Yoshimura M, Yamashita E, Tsukihara T (2008). Structure of human monoamine oxidase A at 2.2-A resolution: the control of opening the entry for substrates/inhibitors. Proc Natl Acad Sci USA..

[19] Groenendyk J, Sreenivasaiah PK, Kim DH, Agellon LB, Michalak M (2010). Biology of endoplasmic reticulum stress in the heart. Circ Res..

[20] Miki T, Miura T, Hotta H, Tanno M, Yano T, Sato T (2009). Endoplasmic reticulum stress in diabetic hearts abolishes erythropoietin-induced myocardial protection by impairment of phospho-glycogen synthase kinase-3beta-mediated suppression of mitochondrial permeability transition. Diabetes..

[21] Yang L, Zhao D, Ren J, Yang J (2015). Endoplasmic reticulum stress and protein quality control in diabetic cardiomyopathy. Biochim Biophys Acta..

[22] Arruda AP, Pers BM, Parlakgul G, Guney E, Inouye K, Hotamisligil GS (2014). Chronic enrichment of hepatic endoplasmic reticulum-mitochondria contact leads to mitochondrial dysfunction in obesity. Nat Med..

[23] Dorn GW, Song M, Walsh K (2015). Functional implications of mitofusin 2-mediated mitochondrial-SR tethering. J Mol Cell Cardiol..

[24] de Brito OM, Scorrano L (2008). Mitofusin 2 tethers endoplasmic reticulum to mitochondria. Nature..

[25] Lewis SC, Uchiyama LF, Nunnari J (2016). ER-mitochondria contacts couple mtDNA synthesis with mitochondrial division in human cells. Science..

[26] Sebastian D, Hernandez-Alvarez MI, Segales J, Sorianello E, Munoz JP, Sala D (2012). Mitofusin 2 (Mfn2) links mitochondrial and endoplasmic reticulum function with insulin signaling and is essential for normal glucose homeostasis. Proc Natl Acad Sci USA..

[27] Lafuente N, Matesanz N, Azcutia V, Romacho T, Nevado J, Rodriguez-Manas L (2008). The deleterious effect of high concentrations of D-glucose requires pro-inflammatory preconditioning. J Hypertens..

[28] Kanasaki K, Taduri G, Koya D (2013). Diabetic nephropathy: the role of inflammation in fibroblast activation and kidney fibrosis. Front Endocrinol..

[29] Richter K, Kietzmann T (2016). Reactive oxygen species and fibrosis: further evidence of a significant liaison. Cell Tissue Res..

[30] Levick SP, Melendez GC, Plante E, McLarty JL, Brower GL, Janicki JS (2011). Cardiac mast cells: the centrepiece in adverse myocardial remodelling. Cardiovasc Res..

[31] Palaniyandi SS, Inagaki K, Mochly-Rosen D (2008). Mast cells and epsilonPKC: a role in cardiac remodeling in hypertension-induced heart failure. J Mol Cell Cardiol..

[32] Larsen CM, Faulenbach M, Vaag A, Volund A, Ehses JA, Seifert B (2007). Interleukin-1-receptor antagonist in type 2 diabetes mellitus. N Engl J Med..

[33] Zhou R, Yazdi AS, Menu P, Tschopp J (2011). A role for mitochondria in NLRP3 inflammasome activation. Nature..

[34] Irwin WA, Bergamin N, Sabatelli P, Reggiani C, Megighian A, Merlini L (2003). Mitochondrial dysfunction and apoptosis in myopathic mice with collagen VI deficiency. Nat Genet..

[35] Rasola A, Bernardi P (2014). The mitochondrial permeability transition pore and its adaptive responses in tumor cells. Cell Calcium..

[36] Malhotra JD, Miao H, Zhang K, Wolfson A, Pennathur S, Pipe SW (2008). Antioxidants reduce endoplasmic reticulum stress and improve protein secretion. Proc Natl Acad Sci USA..

[37] Safiedeen Z, Rodriguez-Gomez I, Vergori L, Soleti R, Vaithilingam D, Douma I (2017). Temporal cross talk between endoplasmic reticulum and mitochondria regulates oxidative stress and mediates microparticle-induced endothelial dysfunction. Antioxid Redox Signal..

[38] Miki T, Yuda S, Kouzu H, Miura T (2013). Diabetic cardiomyopathy: pathophysiology and clinical features. Heart Fail Rev..

[39] Huang ZG, Jin Q, Fan M, Cong XL, Han SF, Gao H (2013). Myocardial remodeling in diabetic cardiomyopathy associated with cardiac mast cell activation. PLoS ONE..

[40] Liu Z, Zhao N, Zhu H, Zhu S, Pan S, Xu J (2015). Circulating interleukin-1beta promotes endoplasmic reticulum stress-induced myocytes apoptosis in diabetic cardiomyopathy via interleukin-1 receptor-associated kinase-2. Cardiovasc Diabetol..

[41] Ni R, Zheng D, Xiong S, Hill DJ, Sun T, Gardiner RB (2016). Mitochondrial calpain-1 disrupts ATP synthase and induces superoxide generation in type 1 diabetic hearts: a novel mechanism contributing to diabetic cardiomyopathy. Diabetes..

[42] Hu Y, Suarez J, Fricovsky E, Wang H, Scott BT, Trauger SA (2009). Increased enzymatic O-GlcNAcylation of mitochondrial proteins impairs mitochondrial function in cardiac myocytes exposed to high glucose. J Biol Chem..

[43] Giorgio V, von Stockum S, Antoniel M, Fabbro A, Fogolari F, Forte M (2013). Dimers of mitochondrial ATP synthase form the permeability transition pore. Proc Natl Acad Sci USA..

[44] Kaludercic N, Giorgio V (2016). The dual function of reactive oxygen/nitrogen species in bioenergetics and cell death: the Role of ATP synthase. Oxid Med Cell Longev..

[45] Hotamisligil GS (2010). Endoplasmic reticulum stress and the inflammatory basis of metabolic disease. Cell..

[46] Lee GH, Lee HY, Li B, Kim HR, Chae HJ (2014). Bax inhibitor-1-mediated inhibition of mitochondrial Ca^2+^ intake regulates mitochondrial permeability transition pore opening and cell death. Sci Rep..

[47] Seferovic PM, Paulus WJ (2015). Clinical diabetic cardiomyopathy: a two-faced disease with restrictive and dilated phenotypes. Eur Heart J..

[48] Koda K, Salazar-Rodriguez M, Corti F, Chan NY, Estephan R, Silver RB (2010). Aldehyde dehydrogenase activation prevents reperfusion arrhythmias by inhibiting local renin release from cardiac mast cells. Circulation..

[49] Zhang Y, Babcock SA, Hu N, Maris JR, Wang H, Ren J (2012). Mitochondrial aldehyde dehydrogenase (ALDH2) protects against streptozotocin-induced diabetic cardiomyopathy: role of GSK3beta and mitochondrial function. BMC Med..

[50] Sturza A, Leisegang MS, Babelova A, Schroder K, Benkhoff S, Loot AE (2013). Monoamine oxidases are mediators of endothelial dysfunction in the mouse aorta. Hypertension..

[51] Buonincontri G, Methner C, Carpenter TA, Hawkes RC, Sawiak SJ (2013). Krieg TMRI and PET in mouse models of myocardial infarction. J Vis Exp..

[52] Buonincontri G, Methner C, Krieg T, Carpenter TA, Sawiak SJ (2014). Functional assessment of the mouse heart by MRI with a 1-min acquisition. NMR Biomed..

[53] O’Connell TD, Rodrigo MC, Simpson PC (2007). Isolation and culture of adult mouse cardiac myocytes. Methods Mol Biol..

