# Supplementary material for: Monoamine oxidase-dependent endoplasmic reticulum-mitochondria dysfunction and mast cell degranulation lead to adverse cardiac remodeling in diabetes
Source: Cell Death Differ. 2018 Feb 19;25(9):1671–85. doi: 10.1038/s41418-018-0071-1 (PMC6015497; doi:10.1038/s41418-018-0071-1)
Supplement: Supplementary file 1 — Supplementary Figures [file 41418_2018_71_MOESM1_ESM.pdf]

Supplementary Information

Supplementary Figure S1. ROS formation in NRVMs treated with HG and pro-inflammatory cytokine IL-1β. Mitochondrial ROS formation determined by fluorescent dye MTR (representative images shown in A) in isolated NRVMs cultured for 48 hours with NG, HG and HM, in the presence or absence of IL-1β, and with or without pargyline **(**data quantification shown in B). Additionally, the effect of IL-1β alone on mitochondrial ROS formation was assessed at different time-points; kinetic measurements were performed up to 1 hour (C), and then at 6, 12 and 24 hours (D). All the experiments were performed in triplicates. Data are expressed as mean ± SEM. Two-way ANOVA test followed by *post hoc* Tukey's multiple comparison test (*$p<0.05$ vs NG vehicle, #$p<0.01$ vs HG vehicle and §$p<0.05$ HG-IL1β vehicle).

Supplementary Figure S2. Relative abundance of MAO-A and –B isoforms in NRVMs vs adult mouse cardiomyocytes. MAO-A and –B protein and gene expression were analyzed by immunoblot (A) and real time RT-PCR respectively, either in NRVMs (B) or adult cardiomyocytes (C).

Supplementary Figure S3. MAO-A protein expression in cardiomyocytes treated with HG and/or IL-1β. MAO-A protein expression was analyzed in NRVMs treated for 48 hours with NG, HG and combination of NG/HG and IL-1β (A), while MAO-B protein expression was analyzed in the adult cardiomyocytes (C) treated as above for 5 hours. MAO-A or -B band intensity was normalized to Red Ponceau staining (B, D, n=3 per each group). Data are expressed as mean ± SEM.

Supplementary Figure S4. Inflammasome activation in NRVMs exposed to HG and/or IL-1β. Robust NLRP3 and ASC upregulation was detected in NRVMs treated with 2 µg/ml lipopolysaccharide (LPS) for 6 hours (A). NLRP3 and ASC expression levels in NRVMs treated

for 48 hours with NG/HG and combination of NG/HG+IL-1β in the absence or presence of pargyline (B). NLRP3 and ASC band intensity was normalized to Red Ponceau staining (n=3 per each group). Data are expressed as mean ± SEM. Two-way ANOVA test followed by *post hoc* Tukey's multiple comparison test (*$p<0.05$ vs NG vehicle).

Supplementary Figure S5. Mitochondrial membrane potential in adult cardiomyocytes exposed to HG and/or IL-1β for 2 hours. Mitochondrial membrane potential was measured in adult cardiomyocytes with the fluorescent dye TMRM following a 2 hour treatment with NG or HG in the absence or presence of IL-1β. Results are expressed as fluorescence ratio before and after the addition of FCCP and normalized to NG. Between 50 and 100 cells were analyzed per condition in each experiment and all the experiments were performed 4 times. Data are expressed as mean ± SEM.

Supplementary Figure S6. Effects of MAO inhibition on tunicamycin and thapsigargin induced cell damage in NRVMs. Gene expression of ER stress markers spliced/total XBP1, ATF4 and CHOP was assessed in NRVMs treated with ER stressors tunicamycin and thapsigargin (A). ROS formation (B) and cell viability (C) were assessed in NRVMs treated with tunicamycin and thapsigargin with or without pargyline. All the experiments were performed in triplicates. Data are expressed as mean ± SEM. Two-way ANOVA test followed by *post hoc* Tukey's multiple comparison test (*$p<0.005$ vs control vehicle, #$p<0.05$ vs tunicamycin vehicle, §$p<0.05$ vs respective control).

Supplementary Figure S7. MAO-B protein expression in heart tissue lysates. MAO-B protein expression was analyzed in heart tissue lysates of control, STZ and STZ+pargyline treated mice after 12 weeks (A). Protein expression was normalized to the expression of GAPDH (B, n=5 per each group). Data are expressed as mean ± SEM.

Supplementary Figure S8. Effect of H_2O_2 on MAO-A and -B activity. Recombinant MAO-A and –B were incubated with 100 μM H_2O_2 for 1 hour. Enzyme activity was measured following administration of tyramine (MAO-A, panel A) or phenylethylamine (MAO-B, panel B). Data are expressed as mean ± SEM.

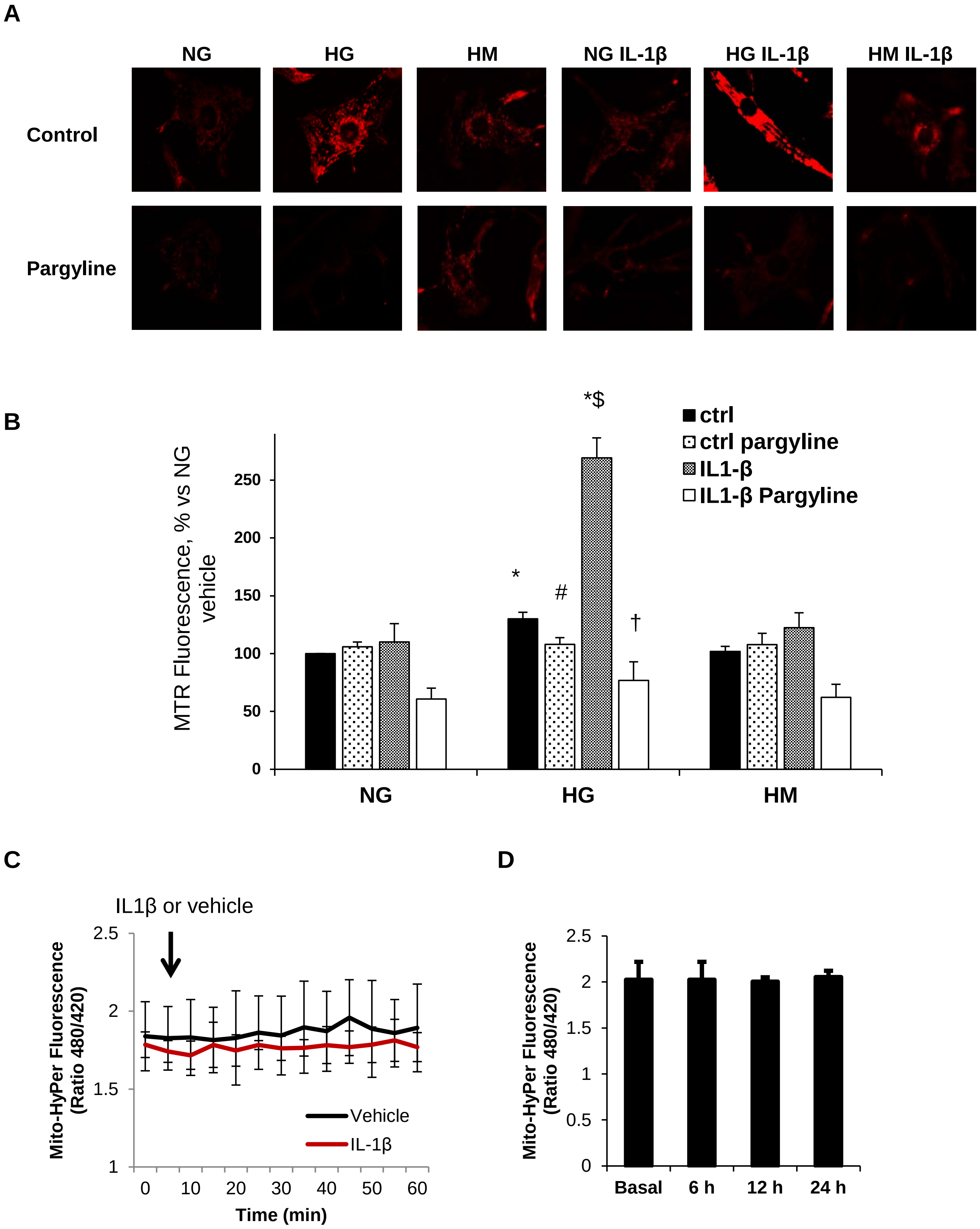

Supplementary Figure S1

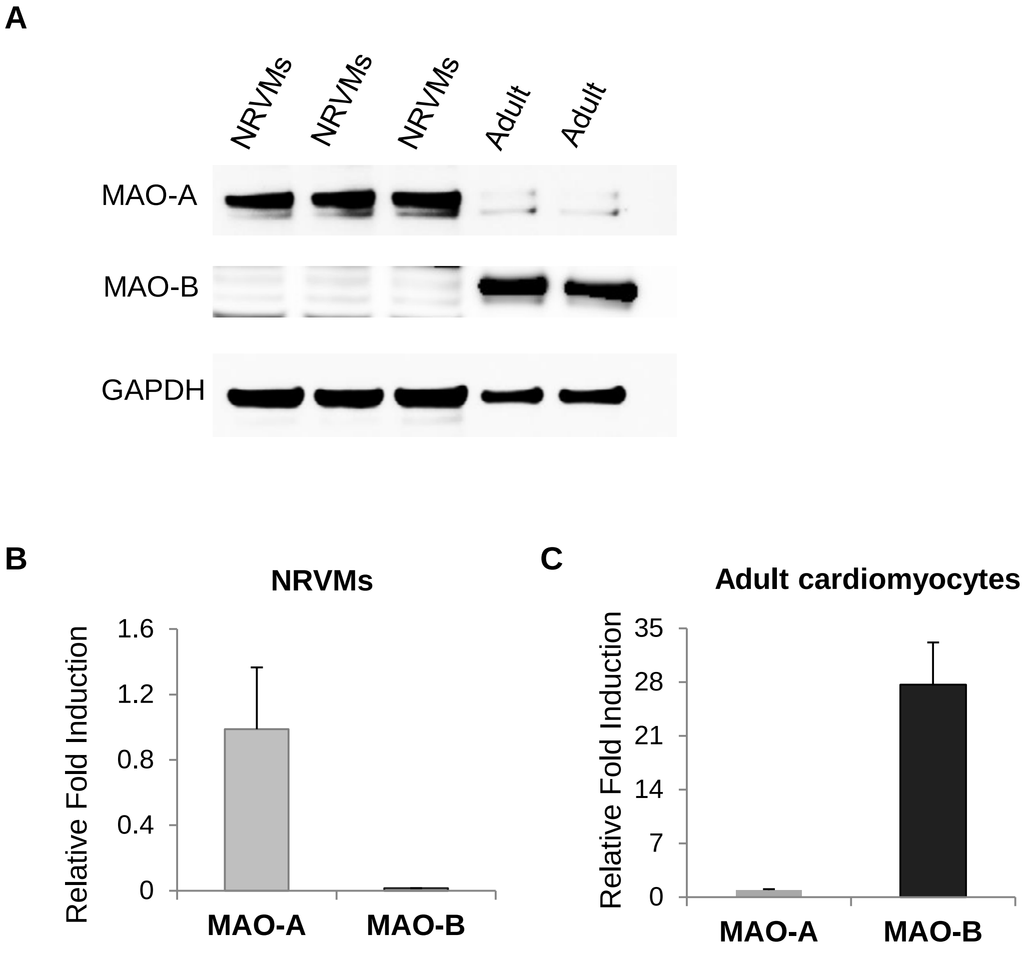

Supplementary Figure S2

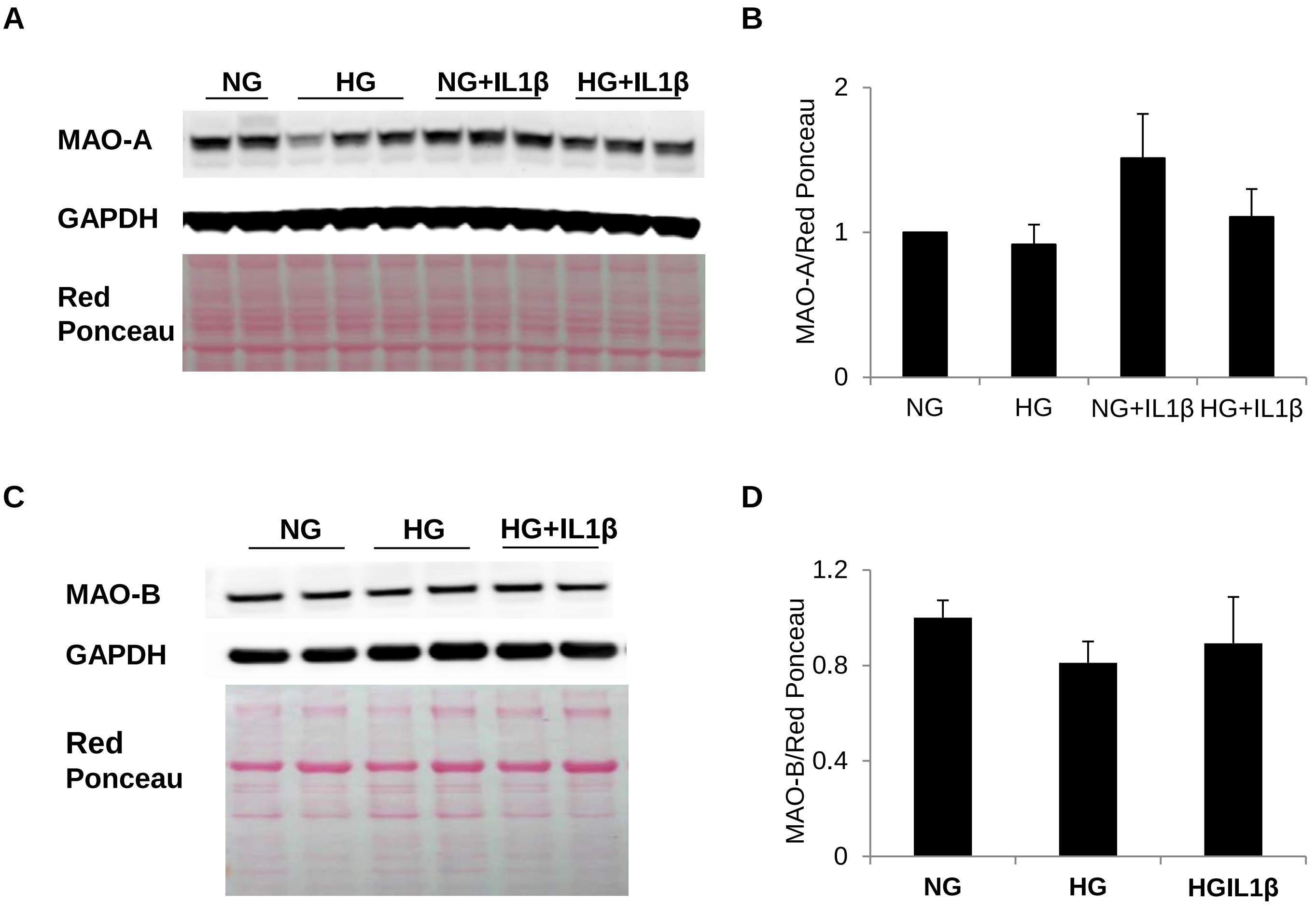

Supplementary Figure S3

A

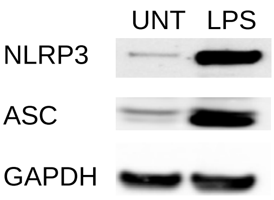

B

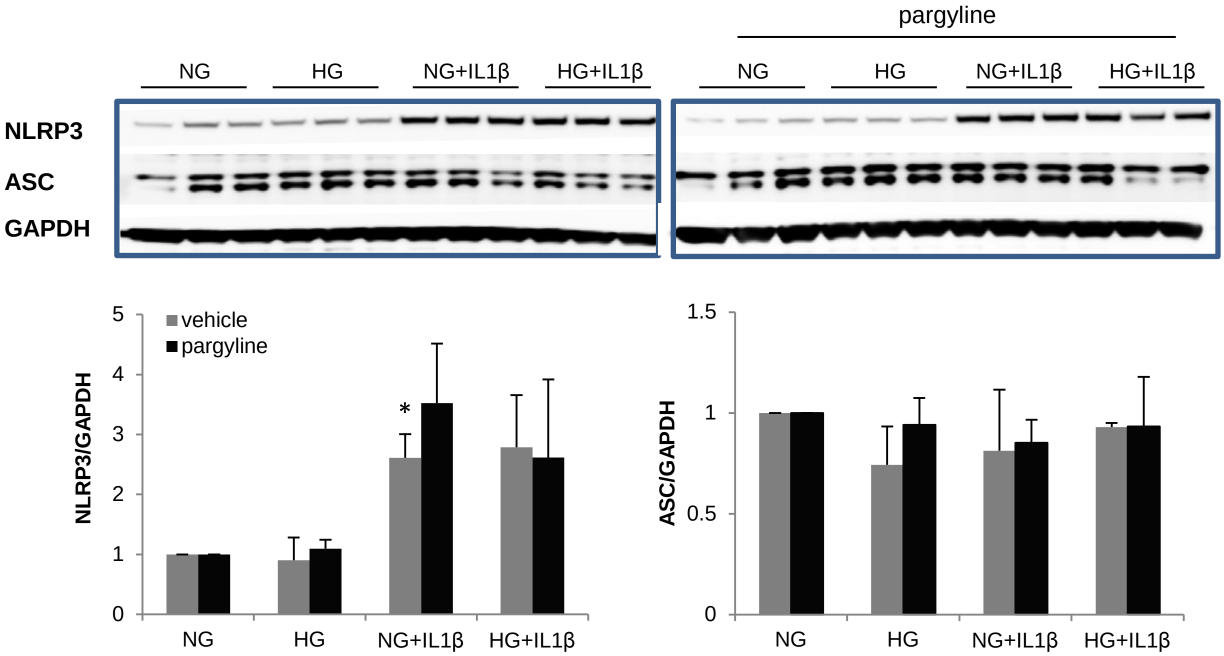

Supplementary Figure S4

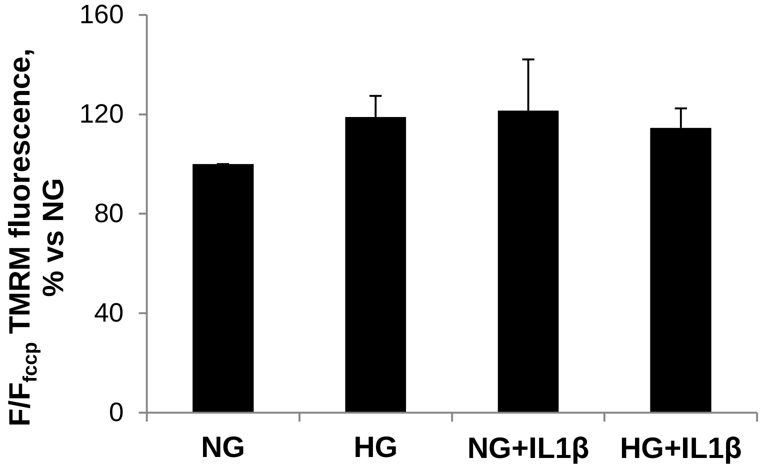

Supplementary Figure S5

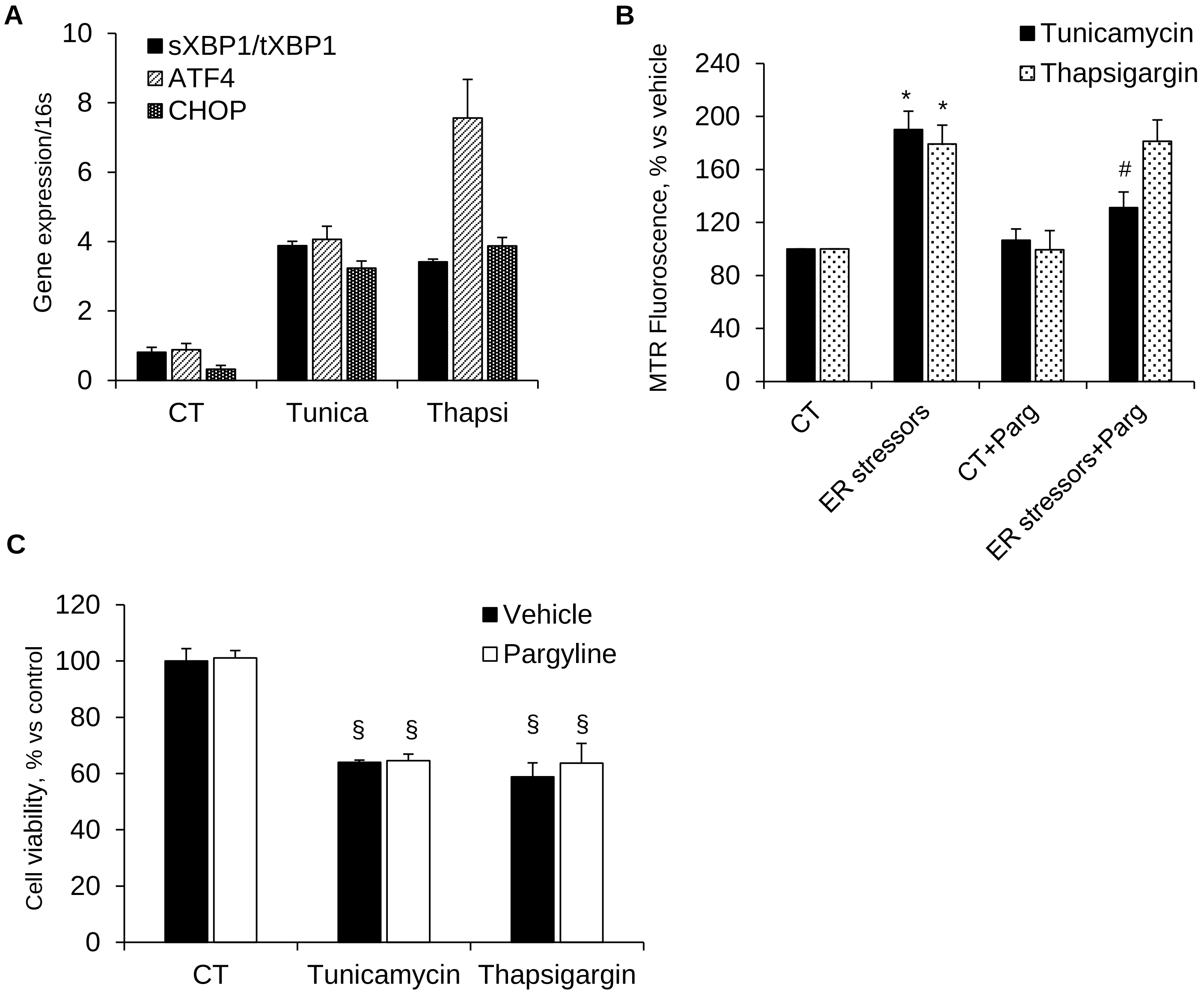

Supplementary Figure S6

A

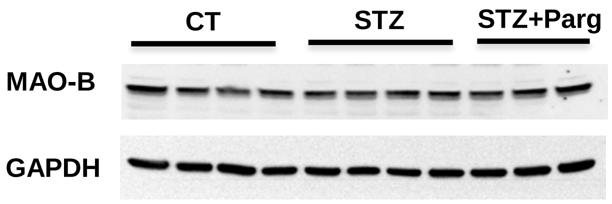

B

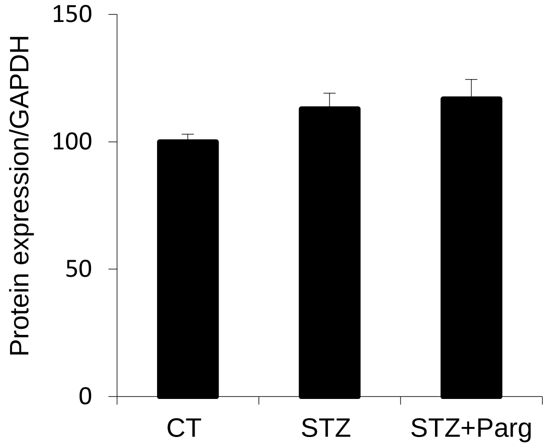

Supplementary Figure S7

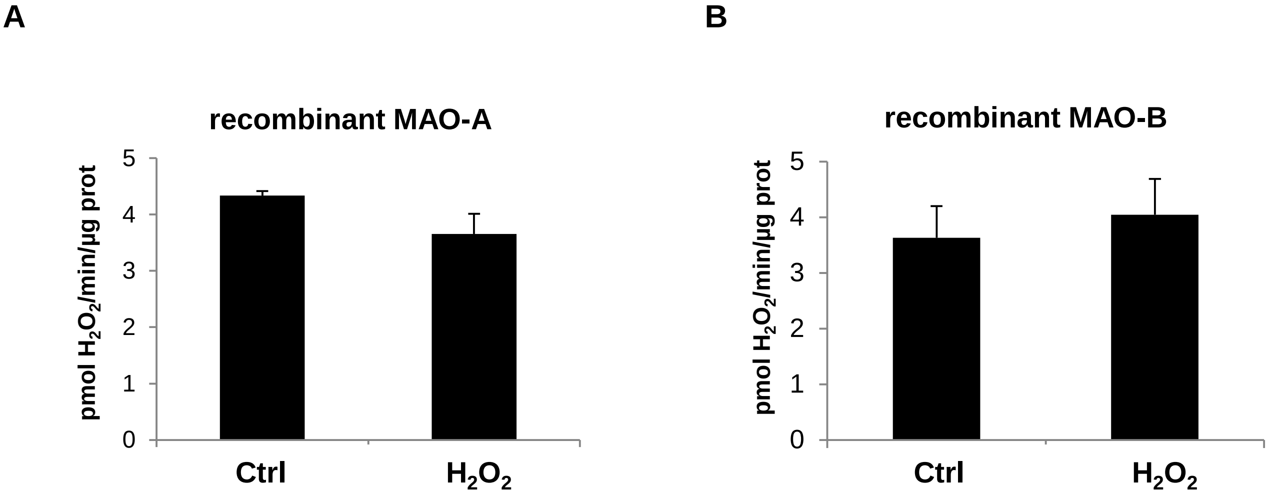

Supplementary Figure S8
